# Xylan utilisation promotes adaptation of *Bifidobacterium pseudocatenulatum* to the human gastrointestinal tract

**DOI:** 10.1038/s43705-021-00066-4

**Published:** 2021-10-28

**Authors:** Yohei Watanabe, Yuki Saito, Taeko Hara, Naoki Tsukuda, Yoshimi Aiyama-Suzuki, Kana Tanigawa-Yahagi, Takashi Kurakawa, Kaoru Moriyama-Ohara, Satoshi Matsumoto, Takahiro Matsuki

**Affiliations:** Yakult Central Institute, Kunitachi, Tokyo Japan

**Keywords:** Bacterial genomics, Microbial ecology, Bacterial evolution

## Abstract

Dietary carbohydrates impact the composition of the human gut microbiota. However, the relationship between carbohydrate availability for individual bacteria and their growth in the intestinal environment remains unclear. Here, we show that the availability of long-chain xylans (LCX), one of the most abundant dietary fibres in the human diet, promotes the growth of *Bifidobacterium pseudocatenulatum* in the adult human gut. Genomic and phenotypic analyses revealed that the availability of LCX-derived oligosaccharides is a fundamental feature of *B. pseudocatenulatum*, and that some but not all strains possessing the endo-1,4-β-xylanase (*Bp*Xyn10A) gene grow on LCX by cleaving the xylose backbone. The *Bp*Xyn10A gene, likely acquired by horizontal transfer, was incorporated into the gene cluster for LCX-derived oligosaccharide utilisation. Co-culturing with xylanolytic *Bacteroides* spp. demonstrated that LCX-utilising strains are more competitive than LCX non-utilising strains even when LCX-derived oligosaccharides were supplied. In LCX-rich dietary interventions in adult humans, levels of endogenous *B. pseudocatenulatum* increased only when *Bp*Xyn10A was detected, indicating that LCX availability is a fitness determinant in the human gut. Our findings highlight the enhanced intestinal adaptability of bifidobacteria via polysaccharide utilisation, and provide a cornerstone for systematic manipulation of the intestinal microbiota through dietary intervention using key enzymes that degrade polysaccharide as biomarkers.

## Introduction

The human gut microbiota (HGM) and host health are interconnected in various manners [1]. Dietary glycan is a major nutrient for the HGM and significantly affects its composition [2]. One feasible strategy for manipulating the HGM is dietary intervention using indigestible carbohydrates that selectively increase endogenous health-promoting microorganisms [3]. Therefore, understanding glycan metabolic ability of specific HGM species could contribute to establishing a systematic means of manipulating gut microbiota. The HGM possesses a wide variety of carbohydrate-active enzymes (CAZymes) used to break down carbohydrates [4, 5], indicating that carbohydrate availability plays an important role in microbiota adaptation to the intestinal environment. Although specific carbohydrate availability influences niche acquisition in the gut of animal models and human infants [6–8], the relationship between carbohydrate availability for individual types of bacteria and their growth remains obscure in the adult human gut, the complex bacterial composition of which widely varies among individuals [9].

Bifidobacteria, which belong to the phylum Actinobacteria, represent one of the core constituents of the HGM [10]. The genus *Bifidobacterium* is characterised by an abundance of genes related to carbohydrate metabolism [11]; thus, glycan metabolism is thought to be important for their adaptation to the intestinal environment [12]. Beyond some uncharacterised species such as *B. pseudocatenulatum*, glycan availability of the major HGM bifidobacterial species has been actively investigated. Infant-dominant taxa (e.g., *B. breve*, *B. bifidum* and *B. longum* subsp. *infantis*) can utilise human milk oligosaccharides (HMO) [13–15], whereas adult-dominant taxa (e.g., *B. adolescentis* and *B. longum* subsp. *longum*) can utilise plant-derived polysaccharides, including starch and the degradation products of long-chain xylans (LCX) [16, 17].

LCX are primary components of various plant cell walls, and are the typical indigestible polysaccharides found in the human diet [18]. LCX consists of a β-1,4-bonded xylose backbone with decorations such as arabinose. In the HGM, a limited number of species of Bacteroidetes and Firmicutes have been known to hydrolyse LCX [19], and the respective degradation mechanisms have been elucidated in detail [20, 21]. Endo-1,4-β-xylanase, belonging to the glycoside hydrolase (GH) 10 family, is a key enzyme that extracellularly cleaves the xylose backbone of LCX, which allows oligosaccharide transporters to take up degradation products such as xylooligosaccharides (XOS) [20, 21]. Parts of short-chain oligosaccharides are released during this process into the extracellular milieu, where they are metabolised by other intestinal bacteria [20, 22]. Therefore, a food web of LCX degradation functions in the human gut, and HGM species with endo-1,4-β-xylanase are generally classified as primary degraders that metabolise LCX and secondary consumers that can only metabolise LCX degradation products.

Bifidobacteria are considered secondary LCX consumers because a strain that can cleave the xylose backbone has not been identified, but several strains can utilise LCX-derived oligosaccharides [22, 23]. However, in this study, we found that some strains of *B. pseudocatenulatum*, an adult-dominant species [10, 24, 25] whose carbohydrate utilisation has not been thoroughly investigated, possess endo-1,4-β-xylanase and could be primary degraders of LCX. Our data revealed the genetic background and ecological significance of a previously overlooked phenotype of bifidobacteria. Furthermore, we confirmed via dietary intervention that LCX availability affects the growth of *B. pseudocatenulatum* in the adult human intestine.

## Materials and methods

### Genome sequencing

We sequenced the genomes of 35 strains of *B. pseudocatenulatum* (Supplementary Table S1). These strains were isolated at the Yakult Central Institute and the species were identified based on the 16S rRNA gene sequence analysis. These strains have been isolated in the course of various studies over the past few decades, including many studies on infants and adults. *B. pseudocatenulatum* cultures were anaerobically incubated in modified Gifu anaerobic medium (Nissui Pharmaceutical, Tokyo, Japan) supplemented with lactose and glucose (both 0.5% wt/vol) at 37 °C for 16 h. These culture conditions were applied throughout the study unless stated otherwise. The detailed procedures for genomic DNA extraction, library preparation for MiSeq (Illumina, San Diego, CA, USA), MinION (Oxford Nanopore Technologies, Oxford, UK) and PacBio RS2 (Pacific Biosciences, Menlo Park, CA, USA), and sequencing are described in the Supplementary Methods.

### Genome assembly, gene prediction and pangenome analysis

We used Unicycler [26] with default parameters for both short-read and hybrid assembly, and Prokka [27] with default parameters for annotating the reconstructed genomes and those downloaded from the RefSeq database. The annotated genomes were then processed with Roary [28] with a default gene identity cut-off parameter of 95% for species level pangenome analysis. A representative sequence from each gene cluster was translated into a protein sequence, and CAZymes were identified using the dbCAN2 server [29]. Proteins were considered CAZymes if they were identified using HMMER, DIAMOND and Hotpep with default parameters. We then built a CAZyme gene distribution matrix (Supplementary Table S2) based on the gene presence-absence table determined using Roary.

### Carbohydrate utilisation assays

Strains of *B. pseudocatenulatum* were cultured until they reached the exponential phase, centrifuged, and then, the resulting pellets were suspended to an OD_600_ of 0.2 in modified peptone yeast extract (PY) medium (100 mM PIPES, pH 6.7, 2 g/L peptone, 2 g/L BBL trypticase peptone, 2 g/L bacto-yeast extract, 8 mg/L CaCl_2_, 19.2 mg/L MgSO_4_ ∙ 7H_2_O, 80 mg/L NaCl, 4.9 mg/L hemin, 0.5 g/L L-cysteine hydrochloride and 100 ng/L vitamin K1). These suspension cultures were inoculated (1% vol/vol) into modified PY medium supplemented with 0.5% (wt/vol) XOS (Xylo-Oligo95P, B Food Science, Aichi, Japan) (PY-XOS), wheat arabinoxylan (Megazyme, Bray, Ireland) (PY-AX) or beechwood xylan (Sigma-Aldrich, Darmstadt, Germany) (PY-XY) and covered with sterile mineral oil (50 μL) to prevent evaporation. Growth was monitored anaerobically by measuring the OD_600_ using a PowerWave 340 plate reader (BioTek, Winooski, VT, USA) every 30 min in an anaerobic chamber for 48 h. The organic acids produced in PY-XY were analysed using high-pressure liquid chromatography as described [8].

### Cloning, expression and purification of recombinant *Bp*Xyn10A

The GH10 domain of the *Bp*Xyn10A gene was amplified by PCR using the primers xynA-GH-F (5’-CATCATCATCATCATGCGGAAGGCGACGCCGTA-3’) and xynA-GH-R (5’-AGCAGAGATTACCTAATCCTTGAATGCGTTCATGC-3’), with the genomic DNA of YIT 11027 as a template. A linearised vector was synthesised by PCR using primers pColdII-F (5’-GTAATCTCTGCTTAAAAGCACAGAATCTA-3’) and pColdII-R (5’-ATGATGATGATGATGATGCACTTTGT-3’), and the pColdII vector (Takara Bio, Otsu, Japan) as a template. These fragments were ligated using In-Fusion HD Cloning Kits (Takara Bio, Otsu, Japan), resulting in pColdII-xynA. *Escherichia coli* BL21 was transformed with pColdII-xynA and cultured to express recombinant *Bp*Xyn10A as described by the manufacturer. Bacterial cells were harvested by centrifugation and lysed with B-PER Bacterial Cell Lysis Reagent (Thermo Fisher Scientific, Waltham, MA, USA) containing lysozyme at 100 µg/mL and 10 U/mL of DNase I. Recombinant *Bp*Xyn10A was further purified using Ni-NTA Spin Column (Qiagen, Hilden, Germany) and analysed by SDS-PAGE.

### Endo-xylanase activity assay

*B. pseudocatenulatum* YIT 11027, YIT 11952 and YIT 4072^T^ cells were grown anaerobically in PY-AX or PY-XOS medium for 16 h. Cultures (1.5 mL) were centrifuged (8000× *g* for 2 min at room temperature); then, supernatants were sterilised by passage through a 0.22-μm filter. Pelleted cells were washed with modified PY medium and resuspended in 1.5 mL of the same medium. The endo-xylanase activity of the supernatant and the cell fractions were assayed using Xylanase Assay kits (XylX6 method) (Megazyme, Bray, Ireland) as described by the manufacturer. According to the manufacturer, this kit is designed to specifically detect only endo-xylanase activity, and not xylosidase or exo-xylanase enzyme activity.

### Purified *Bp*Xyn10A-added culture

*B. pseudocatenulatum* YIT 4072^T^ and *Ba. ovatus* YIT 6161^T^ cells were cultured anaerobically until they reached the exponential phase. Thereafter, cultures (200 μL) were centrifuged (8000× *g* for 2 min at room temperature), then pelleted cells were resuspended in modified PY medium (500 μL), and inoculated (1% vol/vol) into PY-AX medium supplemented with 0, 10, 100 and 1000 ng/mL purified recombinant *Bp*Xyn10A. Growth was monitored anaerobically by measuring the OD_600_ using the PowerWave 340 plate reader.

### RNA-seq analysis

*B. pseudocatenulatum* YIT 11952 was cultured in modified PY medium supplemented with 0.5% (wt/vol) lactose, xylose, XOS, beechwood xylan or arabinoxylan and harvested at mid- to late-log phase. The detailed procedures for total RNA extraction, rRNA removal and sequencing using MiSeq are described in the Supplementary Methods. We obtained a total of 23 million paired-end reads. Low-quality bases (average quality <30) were trimmed off at the 3’, and the resulting reads with N bases, or <70 bp long, were filtered out using cutadapt [30]. Ribosomal RNA reads were removed using SortMeRNA [31]. Filtered reads were then mapped to the complete genome sequence of *B. pseudocatenulatum* YIT 11952 using Bowtie2 [32], and read counts of each gene were determined with featureCounts [33]. The expression levels of each gene were quantified as transcripts per million calculated using Microsoft Excel 2013.

### Batch co-cultures of *B. pseudocatenulatum* and *Ba. ovatus*

The 36 strains of *B. pseudocatenulatum* and *Ba. ovatus* YIT 6161^T^ were separately grown before co-culture. Cells harvested at the mid- to late-log phase were resuspended in modified PY medium adjusted to an OD_600_ of 0.2 (*B. pseudocatenulatum* strains; equivalent to 1 × 10^8.4–9.0^ cells/mL) or 0.02 (*Ba. ovatus*, equivalent to 1 × 10^8.7–9.0^ cells/mL) so that the numbers of cells were similar. Equal amounts of each suspension were inoculated at 2% (vol/vol) into the PY-AX medium. After 48 h of anaerobic co-culture, DNA was extracted using the beads-phenol method as described above. Each sample was analysed by quantitative PCR using an AB7500 real-time qPCR system (Thermo Fisher Scientific, Waltham, MA, USA) to determine cell numbers with specific primers for *B. pseudocatenulatum* (BiCATg-1: 5’-CGGATGCTCCGACTCCT-3’ and BiCATg-2: 5’-CGAAGGCTTGCTCCCGAT-3’) [10] and *Ba. ovatus* (g-Bfra-f: 5’-ATAGCCTTTCGAAAGRAAGAT-3’ and g-Bfra-r: 5’-CCAGTATCAACTGCAATTTTA-3’) [34], respectively.

### Co-culture time-series experiment between *B. pseudocatenulatum* and *B. longum* subsp. *longum*

*B. pseudocatenulatum* YIT 11952 and *B. longum* subsp. *longum* H11-1 (isolated from an infant) were separately grown before co-culture. Cells harvested at the mid- to late-log phase were resuspended in modified PY medium adjusted to an OD_600_ of 0.2. Equal amounts (2% vol/vol) of each suspension were inoculated into 500 μL of PY-AX medium. After 0, 8, 24 and 48 h of anaerobic co-culture, DNA was extracted from subsamples (50 μL) for quantitative real-time PCR using specific primers for *B. pseudocatenulatum* (BiCATg-1 and BiCATg-2) and *B. longum* subsp. *longum* (BiLON-1: 5’-TTCCAGTTGATCGCATGGTC-3’ and BiLON-2: 5’-GGGAAGCCGTATCTCTACGA-3’) [10] using the ABI PRISM 7500 PCR system to determine cell numbers.

### Cereal intervention

#### Experimental design

This study was approved by the ethical committee of the Yakult Central Institute, in accordance with the committee guidelines and the Declaration of Helsinki (2013). This study is registered at UMIN Clinical Trials Registry (number UMIN 000043680). Written informed consent was obtained from 30 Japanese adult participants to participate in this study. Three participants who were prescribed with medications during the experimental period were excluded from data analysis. The experiment was completed by 27 (19 males and 8 females) participants aged 28–65 years who consumed 30 g of All Bran Original wheat bran-rich cereal (Kellogg Japan, Tokyo, Japan) with 180 mL of Accadi lactose-digested milk (Megmilk Snow Brand, Tokyo, Japan) once in the morning and once in the afternoon daily. According to the manufacturer, 60 g of cereal provided approximately 6.6 g of wheat bran arabinoxylan per day. Because almost the entire Japanese population has the genotype for low lactase activity [35], we used 80% lactose-depleted milk according to the manufacturer to minimise the effect of lactose on the growth of *Bifidobacterium* independently of cereal consumption. The experiment comprised pre-intervention (days 1–7), intervention (days 8–14) and post-intervention (days 15–21) periods. Throughout the 21 days of the experiment, the participants were instructed to refrain from consuming fermented milk containing bifidobacteria, prebiotic products and foods rich in LCX, such as other bran-rich cereal, whole grain or brown rice.

Faecal samples were collected on days 4, 7, 11, 14, 18 and 21. To reduce daily individual variations, we averaged the results of individual samples from each period. On days without defecation, faecal samples were collected the next day. If no defecation occurred on days 7, 14 or 21, the previous period was extended until a faecal sample was collected. None of the participants skipped the regime for more than 2 days. Faecal samples collected immediately after defecation were placed in sterile tubes, kept on ice and then stored at −80 °C. Thereafter, DNA was extracted from ten-fold diluted faecal samples using the beads-phenol method as described above.

#### Quantitation of *B. pseudocatenulatum* in faecal samples

*B. pseudocatenulatum* cells were quantified using real-time PCR with a BiCATg primer that targets the 16S rRNA gene. This primer set has been confirmed to be specific for *B. pseudocatenulatum* and *B. catenulatum* and it does not react non-specifically with other bifidobacteria [10]. Although this primer set detects both *B. pseudocatenulatum* and *B. catenulatum* [10], we regarded the measured value as that of only *B. pseudocatenulatum* because none of the participants had amplicon sequence variants (ASVs) close to *B. catenulatum* in the 16S rRNA gene amplicon analysis described below. The PCR mixture (20 μL total volume) contained 1 × TB Green Premix Ex Taq II (Takara Bio, Otsu, Japan), 0.2 μM of each primer and 2 μL of template DNA. The thermocycling conditions used were 94 °C for 10 s, followed by 40 cycles of 94 °C for 20 s, 55 °C for 20 s and 72 °C for 50 s, then a melting-curve programme. Amplification was performed using the ABI PRISM 7500 Real-Time PCR System. For absolute quantification, a standard curve was calculated using a ten-fold dilution series of DNA extracted from the *B. pseudocatenulatum* strain with *Bp*Xyn10A, for which cell numbers were determined. The detection limit for this system was 5.0 × 10^4^ cells/g faeces.

#### Quantification of cells with *Bp*Xyn10A in faecal samples

The numbers of cells with *Bp*Xyn10A were quantified by real-time PCR with *Bp*Xyn10A gene-targeted oligonucleotide primers. To design specific primers, we used the sequences of all *Bp*Xyn10A genes and their homologues obtained from an NCBI blastn search, aligned them, and detected the specific region of the *Bp*Xyn10A gene. Then, we designed pBpXyn10A-F (5’-CGAGAATGCGAACACGTACTTC-3’) and pBpXyn10A-R (5’-CTGCTCGGTGTTGTAATCGTTG-3’), which provided a 94 bp amplicon. Using Primer-BLAST [36], we confirmed that there were no non-specific sequences that could be amplified in the sequences registered in the NCBI nr database. Furthermore, we performed PCR using DNA from the *B. pseudocatenulatum* 35 strains used in our study, and confirmed that specific amplification products were obtained only from strains with the *Bp*Xyn10A gene. The PCR mixture (20 μL total volume) contained 1 × TB Green Premix Ex Taq II (Takara Bio, Otsu, Japan), 0.4 μM of each primer and 2 μL of template DNA. The thermocycling conditions used were 95 °C for 30 s, followed by 40 cycles of 95 °C for 5 s and 60 °C for 34 s, and a melting-curve programme. Amplification was performed using the ABI PRISM 7500 Real-Time PCR System.

#### 16S rRNA gene amplicon analysis

The V1–2 regions of the 16S rRNA gene were amplified. Primer sequence, PCR conditions and details on library preparation are described in the Supplementary Methods. Pooled amplicons were sequenced using the MiSeq platform with the MiSeq reagent kit v2 500 cycle. Obtained reads were analysed using the QIIME2-2020.8 platform [37]. Briefly, the sequence data were denoised using DADA2, and the resulting ASVs were assigned taxonomy in QIIME2 using the SILVA138 database. Alpha-diversity (observed_features, faith_pd and Shannon_index) and beta-diversity (unweighted UniFrac) were analysed using QIIME2. Homology of the ASVs was also analysed using vsearch with the 16S RefSeq nucleotide sequence records to determine the closest species. To analyse species level trends, we grouped ASVs with >97% homology to closely related species as a single species. No ASVs were more closely related to *B. catenulatum* than to *B. pseudocatenulatum*.

### Statistics

Data were statistically analysed using GraphPad Prism 7.0 (GraphPad Software, San Diego, CA, USA). Average CAZyme gene copy numbers of each species in the genome, amounts of organic acids produced in PY-XY medium and numbers of cells with and without *Bp*Xyn10A in the co-culture experiments were compared using Mann–Whitney *U* tests. The effects of the presence or absence of *Bacteroides* species on the growth of *B. pseudocatenulatum* in co-culture experiments, and the increase or decrease of each bacterial species in the cereal intervention study were verified using Wilcoxon signed-rank tests.

## Results

### Composition of *B. pseudocatenulatum* CAZymes

**Fig. 1:**
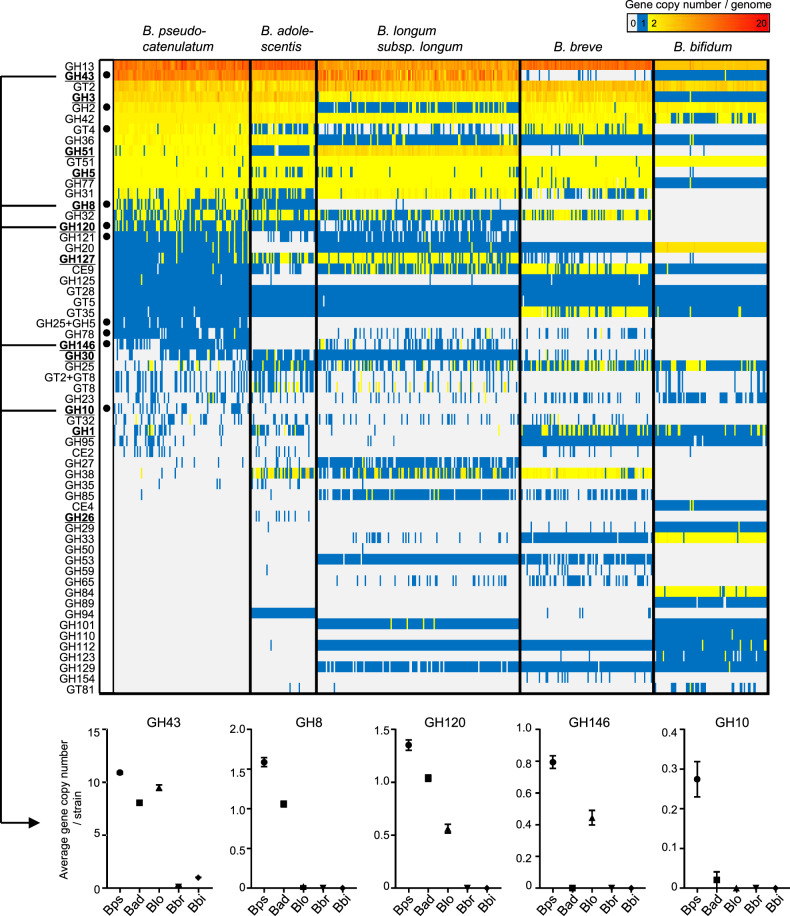
Comparative analysis of CAZyme composition among human-resident bifidobacteria. The heatmap shows copy numbers of the CAZyme gene per genome of 486 strains belonging to *B. pseudocatenulatum* (102 strains), *B. adolescentis* (49 strains), *B. longum* subspecies *longum* (151 strains), *B. breve* (99 strains) and *B. bifidum* (85 strains). Each column represents a strain, and each row represents a CAZyme gene detected from at least one strain in this data set. CAZymes marked in bold and underlined represent xylan degradation-related CAZymes. Black circles indicate the gene with the highest average copy number in *B. pseudocatenulatum*, among other species (Mann–Whitney *U* test; *P* < 0.01). Five of these CAZymes were also shown in separated plots with mean ± standard error. See Supplementary Table S2 for the actual copy number of each CAZyme gene.

To understand the characteristics of carbohydrate utilisation of *B. pseudocatenulatum* within the genus *Bifidobacterium*, we determined draft or complete genomes of 35 isolates of this species (Supplementary Table S1). We then integrated these genomes with 451 publicly available genomes of the major HGM bifidobacteria and compared repertoires of CAZyme genes (Fig. 1 and Supplementary Table S2). We calculated the average number of gene copies for each species and identified ten CAZyme genes that were characteristically abundant in *B. pseudocatenulatum* (Fig. 1). Five of them contained enzymes that were involved in bacterial xylan degradation [38]. Among these, the following three GH families contained enzymes that degrade the xylose backbone of XOS in bifidobacteria: GH43 (xylosidase) [39], GH8 (exo-oligoxylanase) [40] and GH120 (xylosidase) [41]. The two remaining GH families (GH10 and GH146) have not been verified in bifidobacteria; however, GH10 reportedly contains endo-1,4-β-xylanase, which is a key enzyme for LCX degradation in HGM xylanolytic species [42]. Therefore, the availability of oligosaccharides derived from LCX and LCX assimilation might be characteristic phenotypes of *B. pseudocatenulatum*. Conversely, genes encoding CAZymes for HMO utilisation [43] such as GH29 and GH95 (fucosidase), GH33 (sialidase) and GH112 (galacto-N-biose/lacto-N-biose I phosphorylase) were detected in only a few *B. pseudocatenulatum* strains. These data suggest that *B. pseudocatenulatum* has adapted to the environment of the adult human gut, especially those with a diet rich in LCX.

### LCX-related carbohydrate utilisation by *B. pseudocatenulatum*

**Fig. 2:**
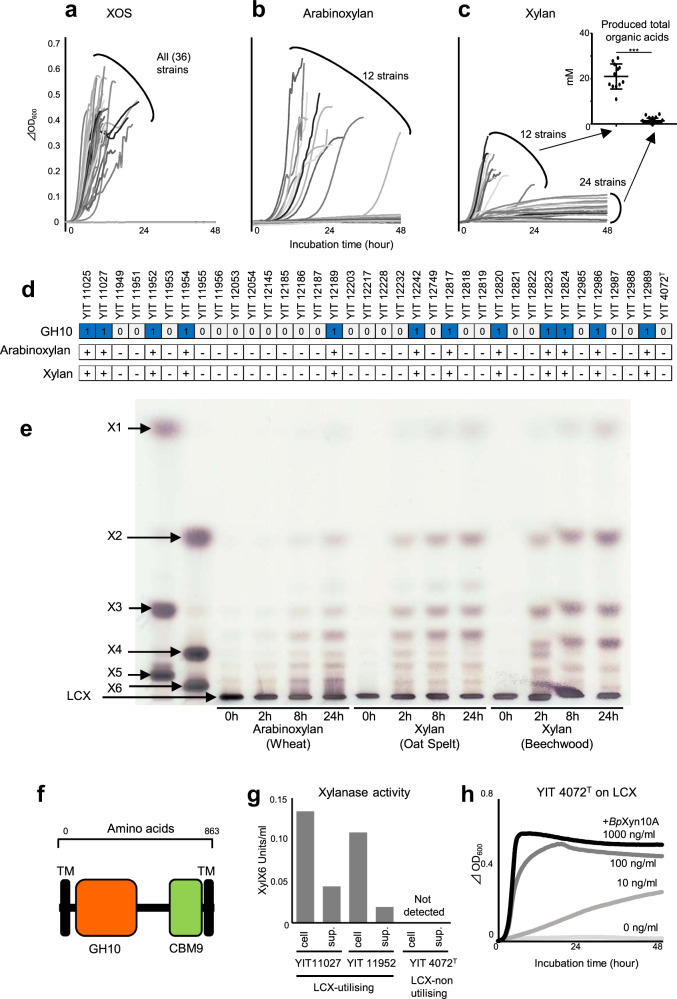
LCX utilisation by the strains that possess a GH10 (*Bp*Xyn10A) gene. Growth curves of our isolates and the type strain in the medium supplemented with XOS (**a**), arabinoxylan (**b**) or xylan (**c**) as the only carbohydrate source. In panel **c**, total organic acid production in the supernatant is also shown. ***P value of Mann–Whitney *U* test <0.001. **d** The association between the presence/absence of predicted GH10 gene and growth phenotype. + indicates an exponential increase in turbidity of >0.1. **e** Endo-xylanase activity of purified recombinant GH10 analysed using thin-layer chromatography. A sample was collected over time after mixing each substrate, and the enzyme was applied. X1: xylose. X2: xylobiose. X3: xylotriose. X4: xylotetraose. X5: xylopentaose. X6: xylohexaose. **f** Domain organisation of *Bp*Xyn10A identified using dbCAN2 [29] and the PSORT web application (http://psort.hgc.jp/form.html); GH10, catalytic module of glycoside hydrolase 10; CBM9, carbohydrate-binding module 9; TM, transmembrane region. **g** Localisation of endo-1,4-β-xylanase activity of the LCX-utilising and non-utilising strains. **h** Growth of the non-LCX-utilising strain (YIT 4072^T^) in the arabinoxylan medium supplemented with multiple concentrations of purified recombinant *Bp*Xyn10A.

We examined the availability of LCX-related carbohydrates in 35 isolates and the type strain of *B. pseudocatenulatum*. All 36 strains grew on a mixture of short-chain XOS (degree of polymerisation, 2–4), indicating that XOS availability is a universal feature of this species (Fig. 2a). Of the 36 strains, 12 could utilise arabinoxylan, a representative LCX (Fig. 2b). Furthermore, the strains that utilised arabinoxylan grew on xylan (Fig. 2c). Although the turbidity of several other strains gradually increased, only these 12 strains produced substantial amounts of organic acids (Fig. 2c), indicating that xylan availability was clearly distinguished between these 12 and other strains. The availability of LCX was completely consistent with the presence of the GH10 gene (Fig. 2d). The GH10 proteins detected in our isolates had 99–100% amino acid sequence similarity among each other. To confirm the enzymatic activity, we prepared a recombinant GH10 domain protein and mixed it with three types of LCX. Because of the accumulation of short-chain oligosaccharides (Fig. 2e), and because all known enzymatic activities in the GH10 family are endo-type (http://www.cazy.org/GH10.html), the enzyme was identified as endo-1,4-β-xylanase, which we named *Bp*Xyn10A. This enzyme contains a GH10 catalytic domain, a carbohydrate-binding module family 9 (CBM9) domain and transmembrane regions (Fig. 2f). Since transmembrane regions were detected, *Bp*Xyn10A was expected to be associated with the membrane, and localise to the cell surface, similar to other HGM xylanolytic species [20, 21]. However, enzyme activity was detected not only on the cell surface but also in the filtered culture supernatant (Fig. 2g). Adding purified recombinant *Bp*Xyn10A allowed a non-LCX-utilising strain to grow on LCX (Fig. 2h). Therefore, LCX availability is determined by the *Bp*Xyn10A gene in the *B. pseudocatenulatum* genome.

### Genetic background of LCX utilisation

**Fig. 3:**
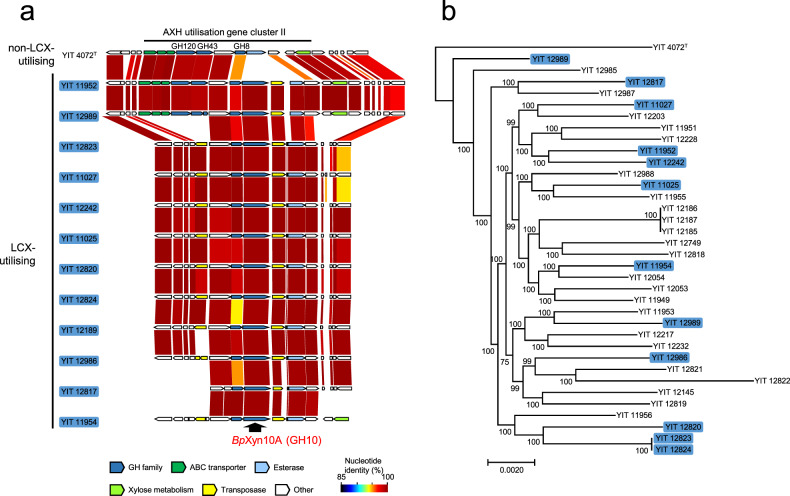
Genetic locus and strain distribution of *Bp*Xyn10A. **a** The gene arrangement around the *Bp*Xyn10A gene. Nucleotide identity of each gene was visualised using GenomeMatcher software [49]. Gene annotation details are also shown in Fig. 4a using the YIT 11952 as an example. **b** A phylogenetic tree based on the alignment of core genes in 35 isolates and the type strain. Strains with the *Bp*Xyn10A gene are shown in blue. Local support values at the branch nodes were computed using the Shimodaira–Hasegawa test with the default parameter settings of fasttree [50].

We analysed the genomic locus of the *Bp*Xyn10A gene to clarify the genetic background of the LCX-utilising strain. The *Bp*Xyn10A gene in all LCX-utilising *B. pseudocatenulatum* strains was located in the arabinoxylan-hydrolysate (AXH) utilisation gene cluster II, which is one of the gene clusters the expression of which is induced by AXH [39] (Fig. 3a). The *Bp*Xyn10A gene was adjacent to the transposase gene in all LCX-utilising strains. These genes were not detected in any other genomic regions of *B. pseudocatenulatum*. Considering that the horizontal transfer of a single transposase gene together with an adjacent gene between bifidobacteria strains in vitro has been confirmed [44], the *Bp*Xyn10A gene might have been horizontally transferred along with a transposase from another taxon. A homology search of public databases showed that the *Bp*Xyn10A was more homologous to proteins in other species of bifidobacteria (maximum, 86.41%) and non-HGM Actinobacteria (maximum, 47.55%) than those detected in Bacteroidetes (maximum, 32.95%) and Firmicutes (maximum, 35.29%) (Supplementary Table S3). These findings indicated that the origin of *Bp*Xyn10A is within the phylum Actinobacteria, but not Bacteroidetes or Firmicutes. However, no genomes with both *Bp*Xyn10A and transposase genes were found in the database; thus, the acquisition source of these genes remains unclear. In phylogenetic tree analysis, strains with *Bp*Xyn10A did not form a single cluster but rather were spread out (Fig. 3b), suggesting that the *Bp*Xyn10A gene was not acquired in a single horizontal transfer event in the past, but may have been acquired at multiple occasions from different genetic sources.

### Expression control of *Bp*Xyn10A

**Fig. 4:**
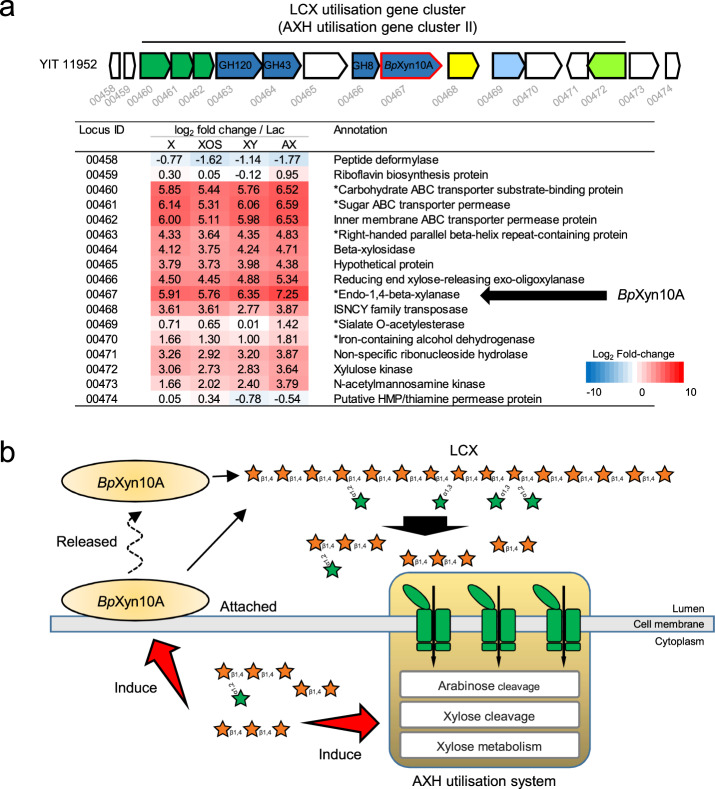
The LCX utilisation system of *B. pseudocatenulatum*. **a** Expression profiles of genes around the *Bp*Xyn10A gene from strain YIT 11952. Heatmap depicts log_2_ fold changes of the expression on xylose (X), XOS, xylan (XY) and arabinoxylan (AX) relative to lactose. Annotation information is from Prokka [27]. Asterisks indicate functional proteins that were not annotated in Prokka, but were found to have >95% amino acid homology by Blastp searches against the NCBI nr database. Locus ID YIT11952_xxxxx are abbreviated with the last numbers after the underscore. **b** LCX utilisation model using arabinoxylan as an example. Initial degradation of LCX occurs by both cell-anchored and extracellularly released *Bp*Xyn10A. Degradation products were imported to the cytoplasm and further degraded by the AXH utilisation system as described previously [39]. Imported degradation products induce the expression of the *Bp*Xyn10A and AXH utilisation system.

In order to assess possible transcriptional regulation of the *Bp*Xyn10A gene, we performed RNA-seq on the LCX-utilising strain cultured with LCX-associated carbohydrates. The expression of *Bp*Xyn10A and its surrounding genes was upregulated in the presence of xylose, XOS, xylan and arabinoxylan in *B. pseudocatenulatum* (Fig. 4a and Supplementary Table S4). A common feature among known HGM xylanolytic bacteria is that intact LCX induces the expression of endo-1,4-β-xylanase [20, 21]. The unique feature of *B. pseudocatenulatum* was that xylose, a monosaccharide, also functions as an induction substrate. The expression induced by LCX-associated carbohydrates was similar to that of the AXH utilisation gene cluster II (Fig. 4a), indicating that this cluster acquired the *Bp*Xyn10A gene without disrupting its original gene regulation. Furthermore, two other AXH utilisation gene clusters were upregulated by LCX (Supplementary Fig. S1). Based on the present and previous findings [39], we propose a model for LCX utilisation by *B. pseudocatenulatum*, in which *Bp*Xyn10A cleaves the xylan backbone and then, resulting oligosaccharides are taken up and further degraded by the AXH utilisation system (Fig. 4b).

### Impact of *Bp*Xyn10A on interspecies interaction

**Fig. 5:**
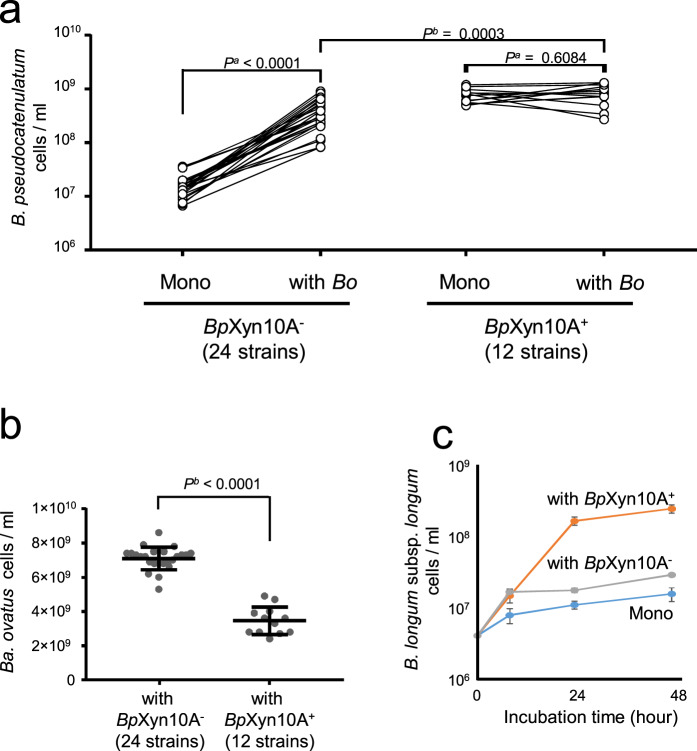
Co-culture of *B. pseudocatenulatum* and other HGM species in medium with arabinoxylan. **a** Cell numbers of mono- and co-cultures of *B. pseudocatenulatum* strains with or without the *Bp*Xyn10A gene. *P*^*a*^: *P* value of Wilcoxon signed-rank test, *P*^*b*^: *P* value of Mann–Whitney *U* test. **b** Cell numbers of *Ba. ovatus* co-cultured with *B. pseudocatenulatum* strains with or without the *Bp*Xyn10A gene. *P*^*b*^: *P* value of Mann–Whitney *U* test. **c** Cell number of *B. longum* subsp. *longum* co-cultured with *B. pseudocatenulatum* strain with (YIT 11952) or without (YIT 4072^T^) the *Bp*Xyn10A gene. Data are expressed as mean of quadruplicate experiments ± standard deviation.

To assess the effect of *Bp*Xyn10A on interspecies interactions, we performed co-culture experiments on medium supplemented with arabinoxylan as the only carbohydrate source. Similar to previous findings of non-LCX-utilising *Bifidobacterium* species [20], the growth of strains without *Bp*Xyn10A was stimulated by co-culture with *Bacteroides ovatus*, an established HGM xylanolytic species [20], whereas that of strains with *Bp*Xyn10A was not (Fig. 5a). The cell numbers of strains with *Bp*Xyn10A in co-cultures were higher than those of the strains without *Bp*Xyn10A (Fig. 5a), indicating that *Bp*Xyn10A provided an ecological advantage even in environments where coexisting bacteria supplied breakdown products. In contrast to *B. pseudocatenulatum*, the numbers of *Ba. ovatus* cells decreased when co-cultured with *B. pseudocatenulatum* strains with *Bp*Xyn10A rather than without this enzyme (Fig. 5b). Furthermore, adding purified recombinant *Bp*Xyn10A reduced the numbers of *Ba. ovatus* cells (Supplementary Fig. S2). Because the expression of xylanase in Bacteroidetes spp. is induced by long-chain XOS but not by short-chain XOS [45], the decreased number of *Ba. ovatus* can be explained by a reduced degree of xylose backbone polymerisation by *Bp*Xyn10A, resulting in a decreased expression of the xylanase. We then examined whether a strain with *Bp*Xyn10A produces a growth substrate for other non-LCX-utilising bifidobacteria. The results of co-culture on arabinoxylan showed that the strain with *Bp*Xyn10A promoted the growth of *B. longum* subsp. *longum* strain H11-1, which utilised XOS but not arabinoxylan (Fig. 5c). Furthermore, we confirmed that the LCX-utilising strain released short-chain oligosaccharides from LCX into the culture supernatant (Supplementary Fig. S3). These results indicate that strains with *Bp*Xyn10A promote the growth of other secondary LCX consumers by releasing breakdown products. Therefore, the presence of the *Bp*Xyn10A gene in the genome defined whether the *B. pseudocatenulatum* strain would act as a keystone species or a recipient of LCX-derived oligosaccharides, and potentially affected the growth of other direct and indirect LCX-degrading microorganisms in the HGM.

### Ecological role of *Bp*Xyn10A in the human intestine

**Fig. 6:**
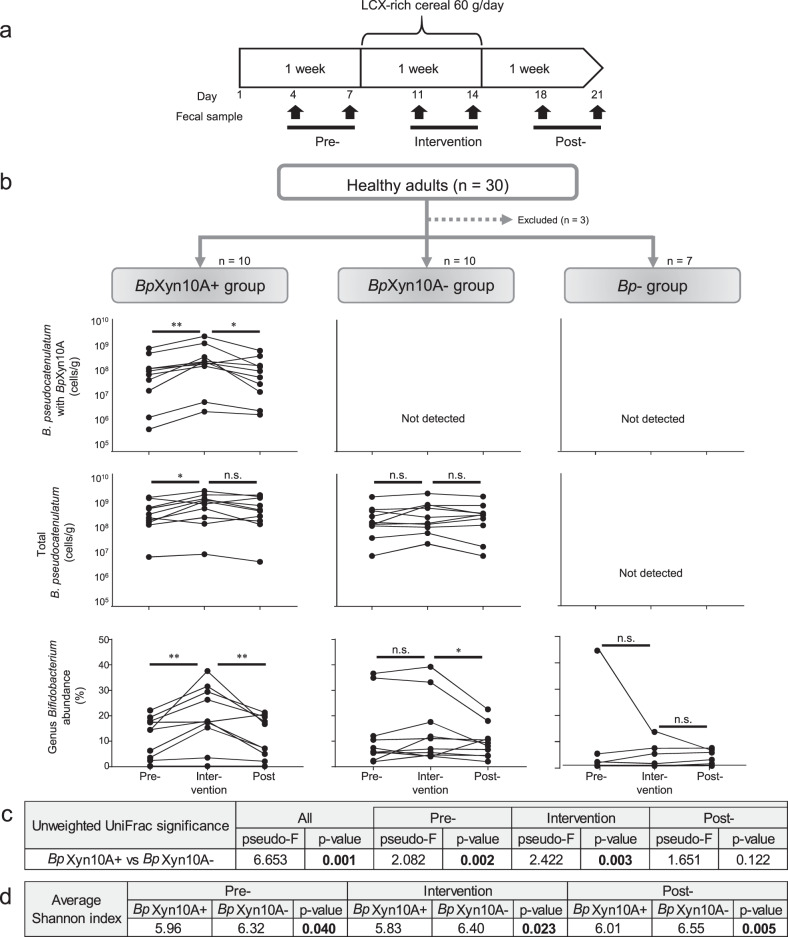
Effect of the presence or absence of the *Bp*Xyn10A gene in the human intestine on the population of *B. pseudocatenulatum* and genus *Bifidobacterium* during LCX-rich cereal intervention. **a** Study design. Participants consumed a 60 g bowl of LCX-rich food per day (see Methods). **b** *B. pseudocatenulatum* cells with *Bp*Xyn10A and total *B. pseudocatenulatum*, and relative abundance of genus *Bifidobacterium* on the participants grouped based on the presence or absence of *B. pseudocatenulatum* and *Bp*Xyn10A gene. Cell numbers were determined using the quantitative value of qPCR targeting the *Bp*Xyn10A and 16S rRNA genes of *B. pseudocatenulatum*. Relative abundance was obtained from 16S rRNA gene amplicon analysis. **P value of Wilcoxon signed-rank test <0.01; *<0.05. n.s.: not statistically significant. **c** PERMANOVA exploring the differences in microbial composition between groups based on unweighted Unifrac distance. **d** Comparison of Shannon index between groups. *P* values of Kruskal–Wallis test are shown.

Based on the findings of our co-culture experiments, we speculated that supplementation with LCX would increase the population of *B. pseudocatenulatum* cells with *Bp*Xyn10A in the human intestine. *B. pseudocatenulatum* has been detected in most Japanese adults [10, 24], and about one-third of our isolates from Japanese adults possesses *Bp*Xyn10A (Fig. 2d). Therefore, we assumed that some Japanese individuals harbour *B. pseudocatenulatum* cells with *Bp*Xyn10A and others harbour those without *Bp*Xyn10A. Under this assumption, we provided Japanese adults with commercially available LCX-rich cereal food made mainly from wheat bran, then compared the behaviour of *B. pseudocatenulatum* cells using qPCR (Fig. 6a). As expected, 10 out of 27 participants (37%) possess *B. pseudocatenulatum* cells with *Bp*Xyn10A (*Bp*Xyn10A+ group), 10 (37%) possess *B. pseudocatenulatum* cells without it (*Bp*Xyn10A– group) and 7 (26%) possess no detectable *B. pseudocatenulatum* (*Bp*– group) (Fig. 6b). The number of cells with *Bp*Xyn10A in the *Bp*Xyn10A+ group ranged from 1 × 10^5.9^ to 1 × 10^9.7^/g faeces (Fig. 6b). The number of cells with *Bp*Xyn10A increased during the intervention in all participants (Fig. 6b). Furthermore, the total number of *B. pseudocatenulatum* cells (regardless of the presence and absence of *Bp*Xyn10A) significantly increased during the intervention in the *Bp*Xyn10A+, but not in the *Bp*Xyn10A– group (Fig. 6b). These findings indicate that *Bp*Xyn10A defines the growth potential of *B. pseudocatenulatum* in LCX-rich intestinal environments.

We then applied 16S rRNA gene amplicon analysis to determine the impact of *Bp*Xyn10A on the composition of HGM. Alpha- and beta-diversity significantly differed between the three groups (Fig. 6c, d and Supplementary Fig. S4a). In particular, the *Bp*Xyn10A+ and *Bp*Xyn10A– groups differed not only during, but also before the intervention, suggesting that *Bp*Xyn10A affected the HGM composition even at the level of LCX supplied in a regular daily diet. The responses of the *Bifidobacterium* genus to the intervention obviously differed among the three groups, with an increase of relative abundance only in the *Bp*Xyn10A+ group (Fig. 6b and Supplementary Fig. S4b). This mainly resulted from the increase of *B. pseudocatenulatum*, and no changes were evident in other bifidobacterial species such as *B. longum* or *B. adolescentis* (Supplementary Fig. S5). The relative abundance of the representative LCX-utilising genera *Bacteroides* and *Roseburia* in the HGM did not significantly increase in either the *Bp*Xyn10A+ or *Bp*Xyn10A– groups (Supplementary Fig. S5). In summary, the ability of *Bp*Xyn10A to promote or inhibit the growth of other bacterial species, such as those found in co-cultures in vitro, was limited in the complex human gut environment. Nevertheless, the results of the 16S rRNA gene amplicon analysis showed that *Bp*Xyn10A affected the growth potential of *B. pseudocatenulatum*.

## Discussion

This study showed that LCX availability is essential to promote adaptation of *Bifidobacterium pseudocatenulatum* in the adult human gut. In infants, HMO availability is known to be a key colonisation factor for bifidobacteria [8], and in adults, LCX availability contributes to niche expansion of the representative adult-dominant species *B. pseudocatenulatum*. This finding extends our understanding of the adaptive strategy of bifidobacteria to the human gut environment. In addition, our findings provide new insights into the food web of LCX degradation in HGM.

We showed that bifidobacteria, which are considered secondary LCX consumers, behave as primary degraders if they possess the *Bp*Xyn10A gene. To the best of our knowledge, the present study is the first to show that HGM organisms other than Bacteroidetes and Firmicutes possess key enzymes for LCX degradation. This leads to the question as to whether LCX metabolism is a characteristic only of *B. pseudocatenulatum* among bifidobacteria. At present, *B. pseudocatenulatum* is the only species expressing biochemically confirmed endo-1,4-β-xylanase in the *Bifidobacterium* genus. Conversely, a *Bp*Xyn10A homologue was also identified in a few strains of other species of HGM *Bifidobacterium* (Supplementary Table S3). These findings suggested that the availability of LCX by HGM *Bifidobacterium* may have been overlooked in previous studies.

The *Bp*Xyn10A gene was integrated into the AXH utilisation gene cluster II (Figs. 3a and 4a), which contains genes related to the uptake and further degradation of oligosaccharides produced by *Bp*Xyn10A, indicating that AXH utilisation gene cluster II has evolved into an LCX-utilisation gene cluster. Because the LCX-utilisation gene cluster was induced by xylose or XOS (Fig. 4a), horizontal transfer of the *Bp*Xyn10A gene generated an LCX-utilisation gene cluster with the regulatory mechanisms for secondary consumers. Because xylose and XOS are products of *Bp*Xyn10A enzymatic activity (Fig. 2e and Supplementary Fig. S3), the LCX-utilisation gene cluster of *B. pseudocatenulatum* can be regarded as having a positive feedback regulation mechanism. This is a feature that is not found in other known LCX-utilisation gene clusters of xylanolytic HGM species, in which intact LCX is the inducible substrate but not low-molecule carbohydrates such as xylose [21, 46, 47]. Having a positive feedback regulation mechanism might explain the obvious increase in *B. pseudocatenulatum* cells with *Bp*Xyn10A during the LCX-rich dietary intervention, even though other representative xylanolytic genera in HGM did not significantly increase.

We showed that *Bp*Xyn10A contributes to increasing numbers of *B. pseudocatenulatum* cells in vitro (Fig. 5) and in the human intestine (Fig. 6). A previous study found that LCX availability is a core fitness determinant in the *Bacteroides* species in mice fed with a high-plant polysaccharide or purified arabinoxylan diet [7]. These findings indicate that LCX availability is a pivotal feature common to different bacterial species among various host gut environments. Therefore, it is reasonable to assume that there is positive selection pressure for *Bp*Xyn10A and that strains with this enzyme will become predominant among the isolates. Nevertheless, strains with *Bp*Xyn10A accounts for only ~33% of our isolates. This discrepancy might be related to the release of *Bp*Xyn10A into the extracellular milieu, where LCX degradation products can be shared by cells producing the enzyme and secondary consumers. Therefore, when an intestinal environment includes cells with *Bp*Xyn10A, negative selection pressure on cells without *Bp*Xyn10A might be reduced. Compared with the membrane-bound endo-1,4-β-xylanase of other HGM xylanolytic species, free *Bp*Xyn10A is more altruistic, especially for secondary LCX consumers.

Although intact LCX has not been considered a substrate for bifidobacteria in the human intestine [22], we found that LCX could be a substrate for the growth of bifidobacteria in the gut of people with detectable *Bp*Xyn10A. Furthermore, during cereal intervention, the number of *B. pseudocatenulatum* cells with *Bp*Xyn10A increased in the entire *Bp*Xyn10A+ group (Fig. 6b). These findings suggest that the *Bp*Xyn10A in faecal samples serve as an accurate biomarker to predict responders, in whom the cell numbers of *B. pseudocatenulatum* would increase under LCX-rich food intervention. Non-responders might also become responders by consuming live *B. pseudocatenulatum* with *Bp*Xyn10A. These possibilities provide a framework that could guide intestinal bifidobacteria recalibration to gain health benefits. In particular, the selective growth of *B. pseudocatenulatum* may contribute to the alleviation of type 2 diabetes [48]. Our study paves the way for systematic manipulation of the intestinal microbiota through dietary intervention using key polysaccharide degradative enzymes as biomarkers.

## Supplementary information

Supplementary figures and methods

Supplementary Tables S1, S2, S3 and S4

## Data Availability

Sequence data that support the findings of this study have been deposited at GenBank and Sequence Read Archive under BioProject accession no. PRJNA745059. The authors declare that all other data supporting the findings of this study are available within the article and its supplementary information files, or from the corresponding author upon request.
