# Supplementary material for: Xylan utilisation promotes adaptation of Bifidobacterium pseudocatenulatum to the human gastrointestinal tract
Source: ISME Commun. 2021 Oct 28;1:62. doi: 10.1038/s43705-021-00066-4 (PMC9723692; doi:10.1038/s43705-021-00066-4)
Supplement: Supplementary file 1 — Supplementary figures and methods [file 43705_2021_66_MOESM1_ESM.pdf]

Supplementary Figures

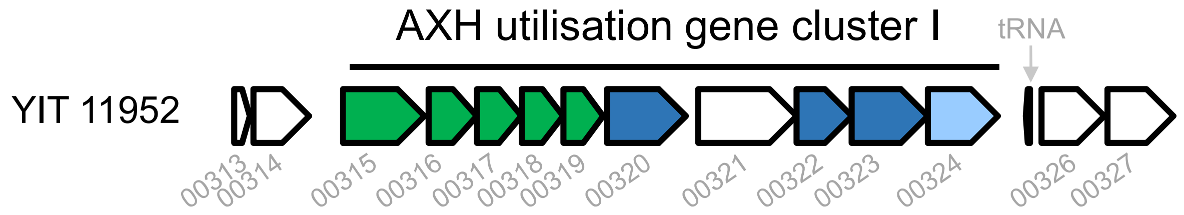

Locus ID	log2 fold change / Lac				Annotation
	X	XOS	XY	AX	
00313	-0.30	-0.20	-0.41	0.21	Ferredoxin
00314	0.38	0.22	0.80	0.54	LL-diaminopimelate aminotransferase
00315	7.31	7.29	7.90	7.24	Glutathione-binding protein GsiB
00316	6.97	6.89	6.92	6.95	Dipeptide transport system permease protein DppB
00317	7.25	7.11	7.11	7.38	Dipeptide transport system permease protein DppC
00318	7.09	6.94	7.08	7.34	Oligopeptide transport ATP-binding protein OppD
00319	7.01	6.91	6.95	7.33	Oligopeptide transport ATP-binding protein OppF
00320	6.60	6.37	6.45	6.82	Beta-xylosidase
00321	5.66	5.54	5.63	6.02	*Right-handed parallel beta-helix repeat-containing protein
00322	5.28	5.17	5.18	5.70	Reducing end xylose-releasing exo-oligoxylanase
00323	4.55	4.48	4.50	5.05	*Arabinofuranohydrolase
00324	4.67	4.51	4.35	5.06	Para-nitrobenzyl esterase
00326	-2.27	-1.90	-2.02	-1.46	DNA polymerase IV
00327	-1.98	-1.62	-1.26	-0.33	CapA family protein

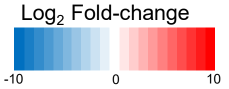

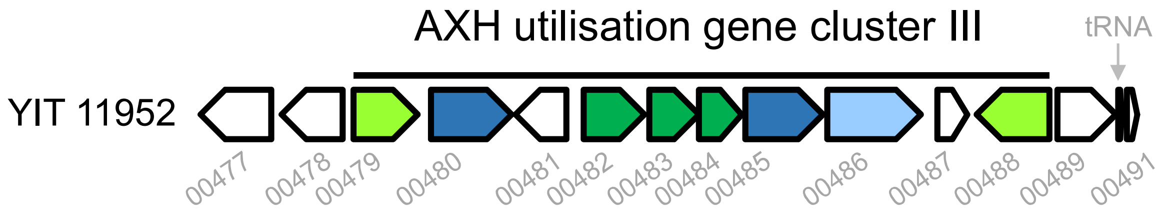

Locus ID	log2 fold change / Lac				Annotation
	X	XOS	XY	AX	
00477	-0.90	-0.82	-1.44	-1.22	putative DNA binding domain-containing protein
00478	1.10	0.91	0.12	0.67	L-methionine gamma-lyase
00479	7.81	7.78	8.01	7.51	Xylose isomerase
00480	4.70	4.53	5.77	3.71	*Glycoside hydrolase family 43 protein
00481	1.90	1.40	2.51	1.68	HTH-type transcriptional repressor CytR
00482	4.59	4.58	5.73	6.17	Multiple sugar-binding protein
00483	4.90	4.91	6.09	6.30	*Sugar ABC transporter permease
00484	4.93	4.76	6.08	6.22	Trehalose transport system permease protein SugB
00485	3.27	3.06	4.61	4.51	Beta-xylosidase
00486	3.10	2.75	4.17	4.08	*9-O-Acetylesterase
00487	0.24	0.28	-0.04	0.10	Putative acetyltransferase
00488	4.07	4.35	4.33	4.57	Xylulose kinase
00489	0.80	0.90	1.09	0.80	N-acetylmannosamine kinase
00491	-0.36	-0.38	-0.79	-1.14	50S ribosomal protein L31

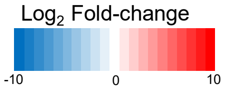

Fig. S1 Gene expression profile of two other AXH utilisation gene clusters.
Expression profiles of the AXH utilisation gene clusters I and III from YIT 11952. Heatmap depicts log_2 fold-changes of the expression of xylose (X), XOS, xylan (XY), and arabinoxylan (AX) relative to lactose. Annotation information is from Prokka (27). Asterisks indicate functional proteins that were not annotated in Prokka, but were found to have >95% amino acid homology in Blastp searches against the NCBI nr database. Locus IDs YIT11952_xxxxx are abbreviated with only the last numbers after the underscore being shown.

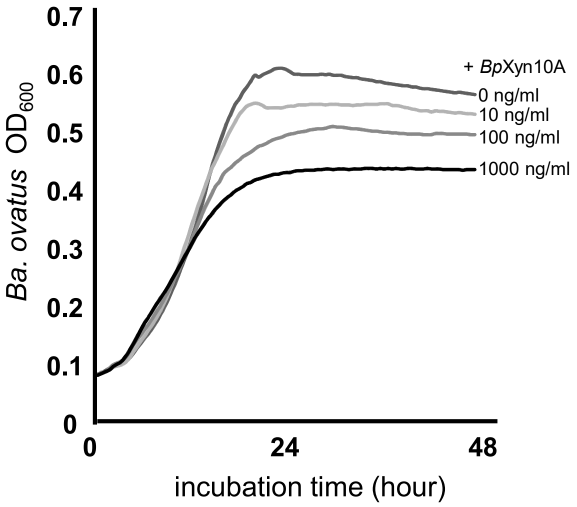

Fig. S2 Growth of *Ba. ovatus* in medium supplemented with arabinoxylan and several concentrations of purified *Bp*Xyn10A.

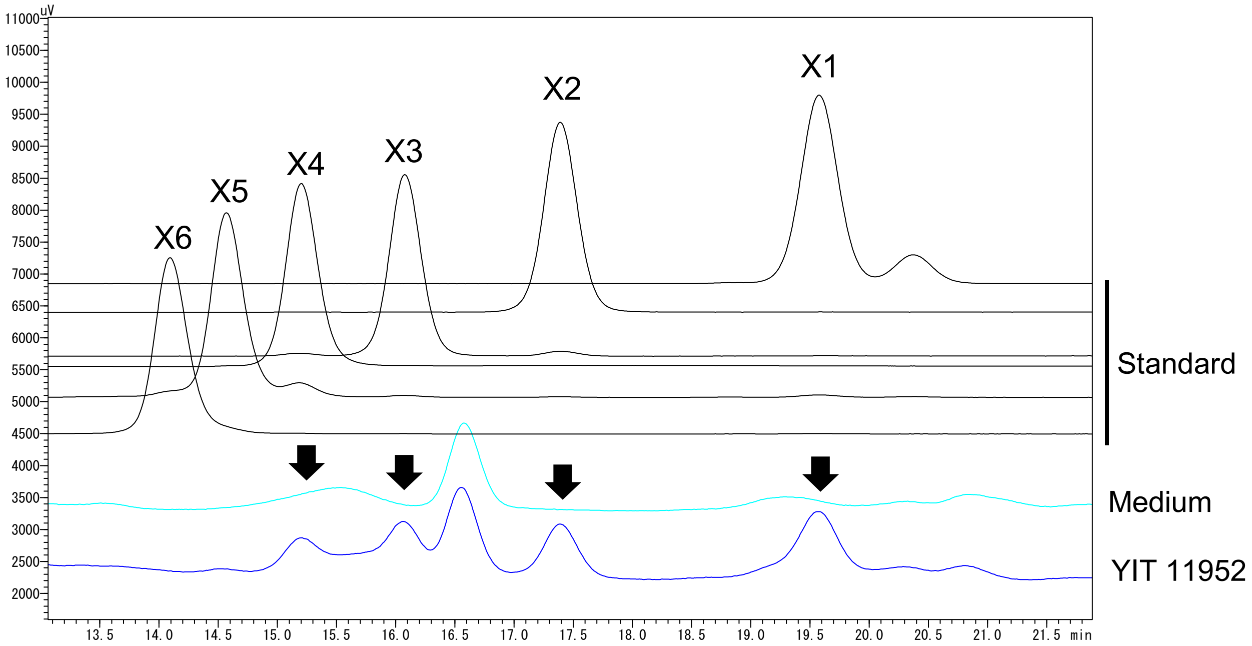

Fig. S3 Oligosaccharides detected by HPLC in the supernatant during the logarithmic growth phase of *Bp*Xyn10A-harbouring strains grown on LCX. X1: xylose. X2: xylobiose. X3: xylotriose. X4: xylotetraose. X5: xylopentaose. X6: xylohexaose. Supernatants obtained from cultures of strain YIT 11952 in PY-XY medium at the logarithmic growth stage were analysed using HPLC (Shimadzu Corporation, Kyoto, Japan) equipped with an RI detector RI-101 (Showa Denko, Tokyo, Japan) and a KS-802 column (Showa Denko, Tokyo, Japan). Black Arrows indicate the detected saccharides.

a

Unweighted UniFrac significance	All		Pre-		Intervention		Post-	
	pseudo-F	p-value	pseudo-F	p-value	pseudo-F	p-value	pseudo-F	p-value
Bp Xyn10A+ vs *Bp* Xyn10A-	6.653	**0.001**	2.082	**0.002**	2.422	**0.003**	1.651	0.122
Bp Xyn10A+ vs *Bp* -	12.703	**0.001**	3.764	**0.001**	4.447	**0.001**	3.368	**0.005**
Bp Xyn10A- vs *Bp* -	6.371	**0.001**	2.117	**0.003**	2.383	**0.002**	4.311	**0.005**

b

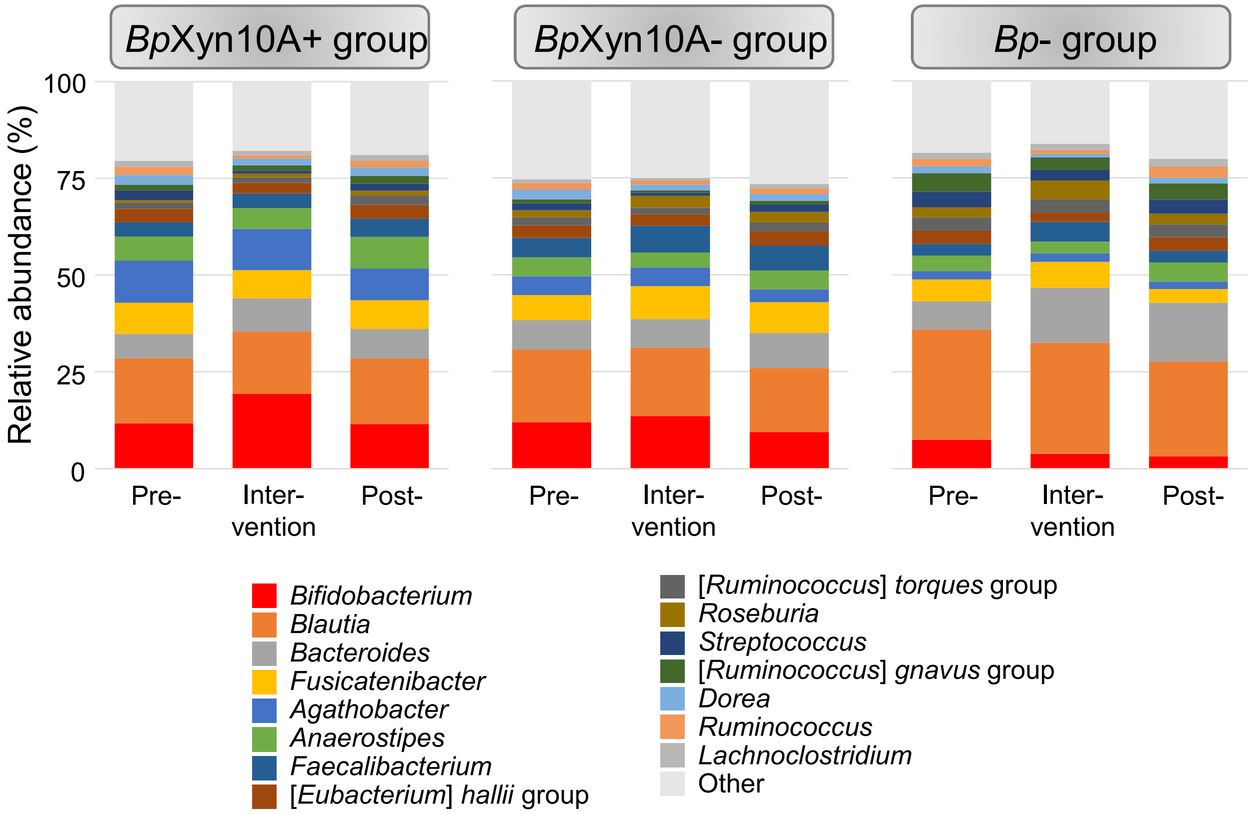

Fig. S4 Comparison of HGM composition between groups. a: PERMANOVA exploring the differences in microbial composition between groups based on unweighted Unifrac distance. **b**: Genus level composition.

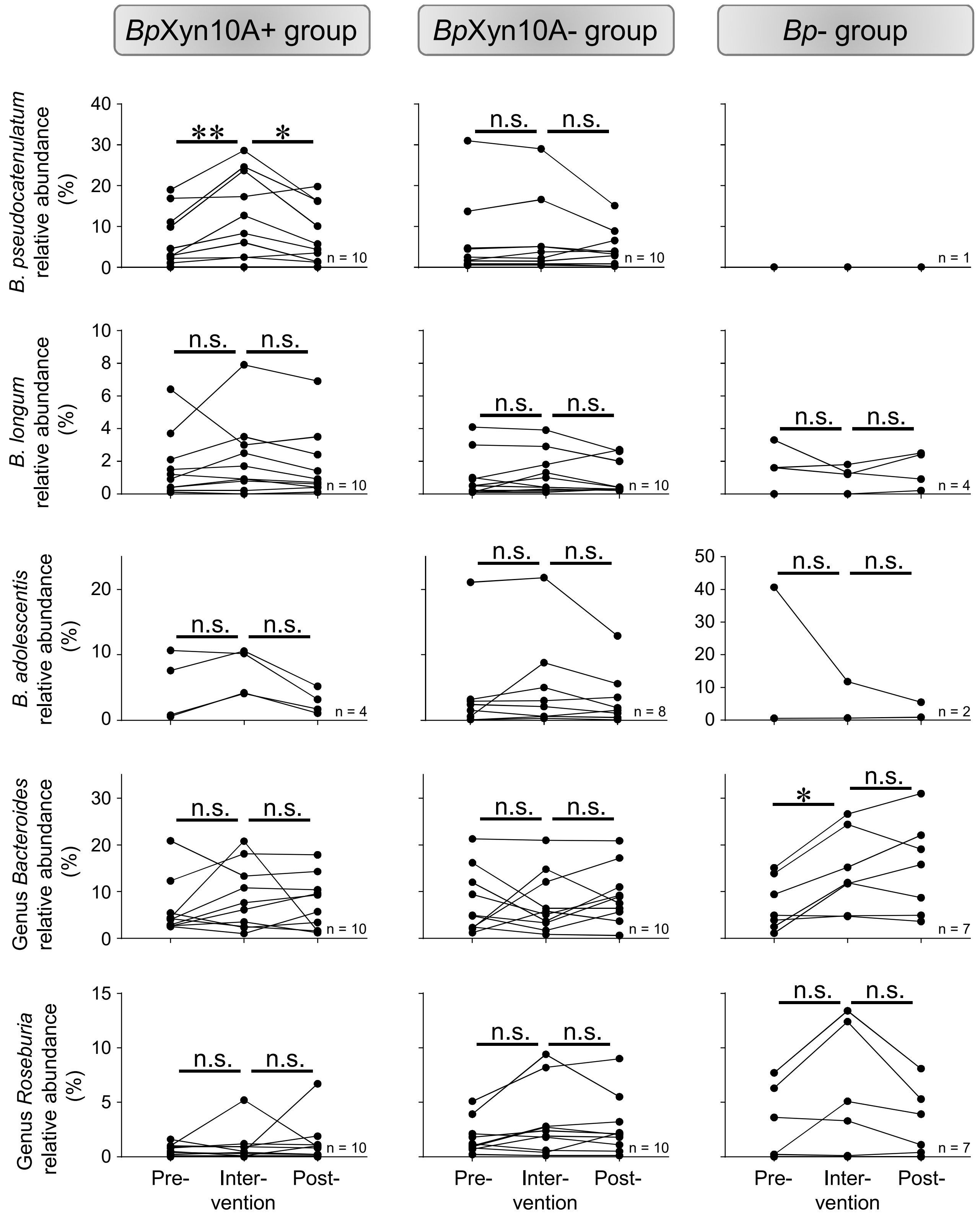

Fig. S5 Responses to LCX-rich food intervention by representative species.

Relative abundance of representative bifidobacterial species and LCX-utilising genera in HGM. A relative abundance >0.1% at least one time point was considered a detection. **: *P*-value of Wilcoxon signed-rank test < 0.01; *: < 0.05. n.s.: not statistically significant.

Supplementary Tables

Table S1 | General features of *B. pseudocatenulatum* genomes determined in this study.

Table S2 | Distribution of CAZyme genes in 486 strains of human-resident bifidobacteria.

Table S3 | Homologous proteins of *Bp*Xyn10A.
NCBI Blastp homology search against the nr database was performed using the amino acid sequence of *Bp*Xyn10A from strain YIT 11027. The origin and homology index of the top 1000 proteins are shown.

Table S4 | TPM values.
Gene expression of *B. pseudocatenulatum* YIT 11952 in response to xylose, XOS, xylan, and arabinoxylan relative to lactose obtained from RNA-seq analysis.

Supplementary Methods

Genome sequencing

Genomic DNA was extracted using the glass beads-phenol method for short-read sequencing. Briefly, cells pelleted from 1.0 mL cultures were suspended in 450 μL extraction buffer (100 mM Tris·HCl, 40 mM EDTA; pH 9.0) with 50 μL of 10% sodium dodecyl sulphate. Glass beads (diameter, 0.1 mm; 300 mg) and 500 μL of phenol were added; then, the mixture was vigorously mixed for 30 s using a FastPrep-24 homogeniser (M.P. Biomedicals, Santa Ana, CA, USA) set at a power level of 6.5. After centrifugation at 15 000 × *g* for 5 min, 400 μL of the supernatant was collected. Subsequently, phenol-chloroform extractions were performed, and 250 μL of the supernatant was subjected to isopropanol precipitation. Finally, the precipitated DNA was suspended in 100 μL of Tris-EDTA buffer. Genomic libraries were constructed using Nextera XT DNA Sample Prep Kits (Illumina, San Diego, CA, USA). Paired-end sequencing proceeded using the MiSeq platform with the MiSeq reagent kit v2 500 cycle (Illumina, San Diego, CA, USA). For long-read sequencing, genomic DNA was extracted using NucleoBond buffer set III and NucleoBond AXG 20 (Macherey-Nagel, Düren, Germany) as described by the manufacturer. Nanopore libraries were prepared using rapid or ligation sequencing kits (Oxford Nanopore Technologies, Oxford, UK), and sequenced using a MinION device with a FLO-MIN 106 flow cell (Oxford Nanopore Technologies, Oxford, UK) as described by the manufacturer. PacBio libraries were prepared using the SMRTbell template prep kit 1.0 (Pacific Biosciences, Menlo Park, CA, USA), and sequenced using a PacBio RS2 system (Pacific Biosciences, Menlo Park, CA, USA) as described by the manufacturer.

RNA-seq analysis

B. pseudocatenulatum YIT 11952 was cultured in modified PY medium supplemented with 0.5% (wt/vol) lactose, xylose, XOS, beechwood xylan, or arabinoxylan and harvested at mid- to late-log phase. Total RNA was extracted using RiboPure Bacteria RNA Purification Kits (Thermo Fisher Scientific, Waltham, MA, USA) as described by the manufacturer. Ribosomal RNA was removed using the Ribo-Zero rRNA Removal Kit (Illumina, San Diego, CA, USA). Sequencing libraries were constructed by using TruSeq Stranded mRNA Sample Prep Kits (Illumina, San Diego, CA, USA) as described by the manufacturer, and high-quality, alignable sequence data were obtained by paired-end sequencing using MiSeq Reagent V3 Kits (150 cycles) and the MiSeq sequencing platform.

Cereal intervention

16S rRNA gene amplicon analysis

The V1–2 regions of the 16S rRNA gene were amplified using a forward 27Fmod2-MiSeqV2 primer (5’-AATGATACGGCGACCACCGAGATCTACACTCTTTCCCTACACGACGCTCTTCCGATCT-AGRGTTYGATYMTGGCTCAG-3’) and a reverse 338RMiSeqV2 primer (5’-CAAGCAGAAGA CGGCATACGAGAT-NNNNNNNNNNNN-GTGATGGAGTTCAGACGTGTGCTCTTCCGATCT-GCTGCCWC CCGTAGGWGT-3’). A unique 12-base Golay barcode (Ns) preceded the primers for sample identification. The PCR mixture (50 μL total volume) contained 1 × TB Green Premix Ex Taq II, 0.1 μM of each primer, and 1 μL of template DNA. The thermocycling conditions used were 95 ° C for 30 s, followed by 27 cycles of 95 ° C for 5 s, 55 ° C for 30 s, and 72 ° C for 40 s. Amplification was performed using the ABI PRISM 7500 Real-Time PCR System. Amplicons were purified using an AMPure XP Kit (Beckman Coulter, Brea, CA, USA) and quantified using a Quant-iT PicoGreen dsDNA Kit (Thermo Fisher Scientific, Waltham, MA, USA). Pooled amplicons were sequenced using the MiSeq platform with the MiSeq reagent kit v2 500 cycle.
